# The complete chloroplast genome sequence of *Ulmus parvifolia* (Ulmaceae)

**DOI:** 10.1080/23802359.2020.1791006

**Published:** 2020-07-24

**Authors:** Manyu Li, Qingyang Chen, Lin Zhang, Peng Guo, Yihan Wang

**Affiliations:** a College of Life Sciences, Henan Agricultural University, Zhengzhou, China; b College of Forestry, Henan Agricultural University, Zhengzhou, China

**Keywords:** *Ulmus parvifolia*, chloroplast genome, Ulmaceae, phylogenetic analysis

## Abstract

*Ulmus parvifolia* Jacq is a kind of landscape tree endemic to East Asia. In this study, the complete chloroplast genome of *U. parvifolia* was sequenced. The genome was 159,259 bp in length, with a large single-copy (LSC) region of 88,451 bp, a small single-copy (SSC) region of 19,598 bp, and two inverted repeat (IR) regions of 25,605 bp, each. The genome consisted of 121 genes, including 77 protein-coding genes, 36 tRNA genes, and 8 rRNA genes. The phylogenetic results indicated that *Ulmus* is not monophyletic and *U. parvifolia* constitute a well-supported clade sister to *U.* Sect. *Ulmus*. The complete chloroplast genome of *U. parvifolia* will provide important information for phylogenetic and evolutionary studies in Ulmaceae, as well as the other closely related families.

*Ulmus* L. is a genus of trees in family Ulmaceae, including 40 species and distributed in the Northern Hemisphere (Wu et al. 2003). *Ulmus parvifolia* is useful for a multitude of landscapes, as ornamental shade trees, which can survive well in climate extremes and resistant to Dutch elm diseases (Thakur and Karnosky 2007). Nevertheless, its genetic background and resources have not been widely studied. In this study, we reported the complete chloroplast genome sequence of *U. parvifolia* for future phylogenetic and genetic studies (GenBank accession number: MT604161).

A single individual of *U. parvifolia* was used as a sampling object from the Fudian Village (114°49′46′′ E, 31°50′12′′ N) in Xinyang City, Henan Province, China, and the voucher specimens were deposited at the Herbarium of Henan Agricultural University (voucher number ljm-20-0518). Total genomic DNA was extracted following the modified CTAB protocol (Yang et al. 2014), then sequenced using the Illumina HiSeq2500 platform. The generated reads were assembled by GetOrganelle v1.5.2 (Jin et al. 2018) with *Ulmus pumila* (GenBank number: KY244086) as the reference. The genome annotation was performed with CpGAVAS webserver (Liu et al. 2012), then the boundaries between inverted repeats were confirmed manually using Geneious v9.12 (https://www.geneious.com/). The physical map of the chloroplast genome was drawn with OGDRAW v1.3.1 (Lohse et al. 2007).

The chloroplast genome of *U. parvifolia* is 159,259 bp in length, and the structure was a typical quadripartite, which exhibited a large single-copy region (LSC) with 88,451 bp, a small single-copy region (SSC) with 19,598 bp, and two inverted repeat (IR) regions of 25,605 bp, each. The base compositions of *U. parvifolia* chloroplast genome included 31.2% A, 18.0% G, 18.7%C, 32.1% T, with an overall GC content of 35.6%. The circular genome contained 121 genes, including 77 protein-coding genes, 36 tRNA genes, and 8 rRNA genes. Complete chloroplast genomes from Ulmaceae (17 species) and other related families (*Morus mongolica*, *Ficus racemosa*, *Broussonetia papyrifera*) were chosen as outgroups in the phylogenetic analysis. Sequence alignment was conducted by MAFFT v7.450 (Katoh and Standley 2013), and a phylogenetic tree was generated using RAxML v.8.1.2 (Stamatakis 2014) based on maximum-likelihood methods and the GTRGAMMA nucleotide substitution model by 1000 bootstrap replicates. Phylogenetic analysis indicated that *Ulmus* may be paraphyletic with *Zelkova* nested within it. Our results informally recognized the four subgeneric sections (Sect. *Blepharocarpa*, Sect. *Chaetoptelea*, Sect. *Ulmus* and Sect. *Microptelea*) within *Ulmus*, which was in accordance with previous studies (Han et al. 2011; Liu et al. 2019; Zhang et al. 2019). The focal species *U. parvifolia* constitute a clade sister to the six species of *U*. Sect. *Ulmus*. *Ulmus americana* from Sect. *Blepharocarpa* and *Ulmus elongata* from Sect. *Chaetoptelea* are more closely related to *Zelkova* than the rest congeneric species included (Figure 1). This study was the first report on the complete chloroplast genome of *U. parvifolia*, which would be beneficial to potential studies on phylogenetics of the genus and related group in Ulmaceae.

**Figure 1.**
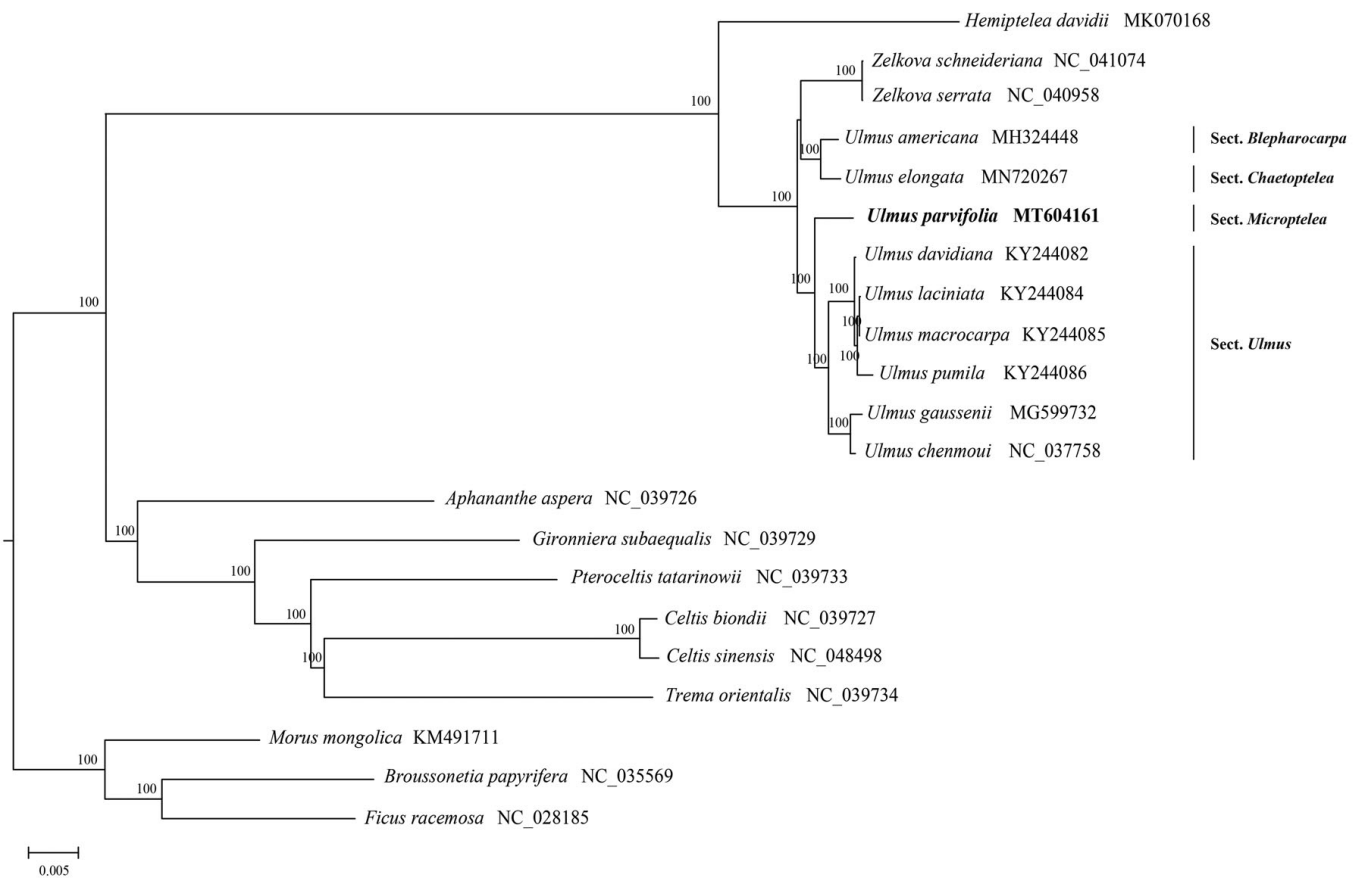
Phylogenetic tree reconstruction of 21 taxa with maximum-likelihood method based on complete chloroplast genomes sequences. Bootstrap values based on 1000 replicates were provided near branches. *Ulmus parvifolia* is highlighted in bold.

## Data Availability

The data that support the findings of this study are openly available in the National Center for Biotechnology Information (NCBI) at https://www.ncbi.nlm.nih.gov/, reference number MT604161.
